# Amyloïdosis, sarcoidosis and systemic lupus erythematosus

**DOI:** 10.11604/pamj.2016.24.23.8853

**Published:** 2016-05-06

**Authors:** Amel Rezgui, Imene Ben Hassine, Monia Karmani, Fatma Ben Fredj, Chadia Laouani

**Affiliations:** 1 Internal Medicine Department, CHU Sahloul, Tunisia

**Keywords:** Amyloïdosis, sarcoïdosis, lupus erythematosus

## Abstract

The occurrence of renal and multiple organ Amyloïdosis is currently considered exceptional in the course of systemic lupus erythematosus. We report a case of a concomitant SLE and Amyloïdosis in a 57 year old female patient with hypothyroidism history, who presented with erythema nodosum, fever, arthralgia and sicca syndrome. Biological findings showed an inflammatory syndrome, renal failure, proteinuria (1g / 24h), positive auto antibodies and anti DNA. Lung radiology revealed medistinal lymphadenopathy, pleural nodules, ground glass infiltrates and pleuritis. Bronchial biopsy showed non specific inflammation. The salivary gland biopsy showed amyloïd deposits. This case report reminds us that lupus and Amyloïdosis association, although exceptional remains possible. The occurrence of Lofgren syndrome in this situation make the originality of this report.

## Introduction

Systemic lupus erythematosus (SLE) is characterized by a large clinical polymorphism which explains the diagnostic difficulties encountered in some cases. Amyloidosis is histologically diagnosed by the presence of insoluble protein deposits in tissues. The AA amyloid form is secondary to a chronic inflammatory condition due to systemic diseases. The occurrence of renal and multiple organ amyloidosis is exceptional in the course of systemic lupus erythematosus. We report a case of concomitant diagnosis of SLE and amyloidosis.

## Patient and observation

**Figure 1:**
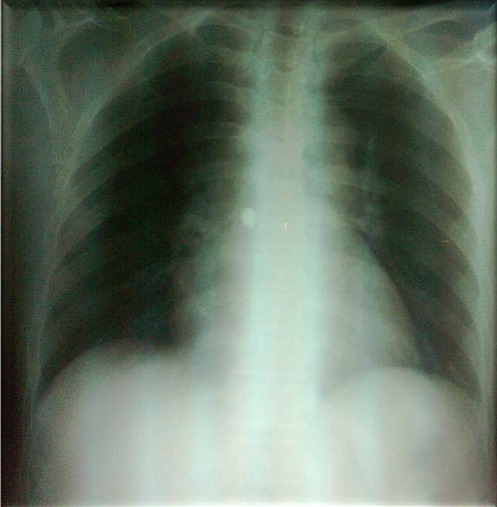
Chest X-ray showing Mediastinal enlargement

**Figure 2:**
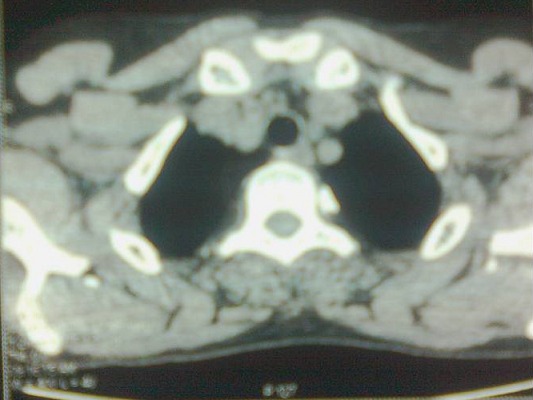
Mediastinal lymph nodes in Barety space and bilateral hilar lymphadenopathy on chest CT

**Figure 3:**
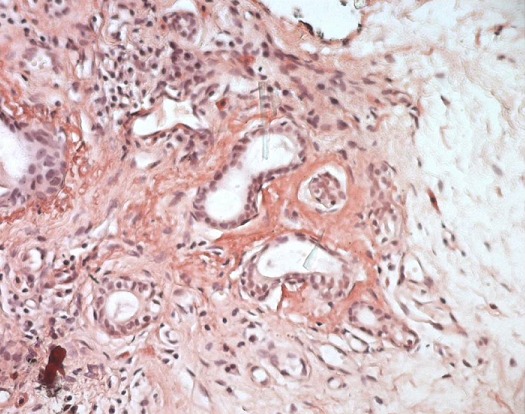
Congo red stain: amyloid deposits around the vessels and interlobular ducts

**Figure 4:**
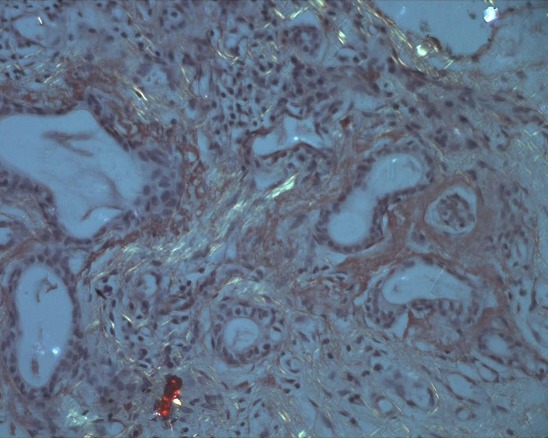
Irefringent amyloid deposits under polarized light

This is a 57 year old female with hypothyroidism history for 20 years who consulted for fever, arthralgia and erythema nodosum. She presented with a 10 day history of subcutaneous nodules in lower limbs, fever, and arthritis. On physical examination, there was hepatosplenomegaly, and no lymphadenopathy. On the skin examination there was hot nodules on the front and sides of the legs. On ophtalmological examination, there wa keratitis. Biological findings showed an elevated erythrocyte sedimentation rate (123 mm during the first hour), a normocytic normochromic non-regenerative anemia (9 g / dl), a positive polyclonal hyper gamma globulin rate (16 g / L), an elevated C-reactive protein rate (190 mg / L), renal failure (creatinine 200 umol / l, Urea 16 mmol / l, Renal clearance: 28 ml / min), anti-nuclear and anti-native DNA antibodies were positive. There was also significant proteinuria (1g/day). Calcium and phosphate balance over a 3 day periods was normal. Chest X-ray showed Mediastinal enlargement suggestive of mediastinal adenomegaly with calcifications (Figure 1). On chest CT there was mediastinal lymph nodes in Barety space and bilateral hilar lymphadenopathy which were partially calcified. There were changes in the lung parenchyma described as ground glass infiltrates with pleural nodules, pleural and pericardial effusions (Figure 2). Tuberculin intradermal test was negative, mycobacteruim tuberculosis wasn't present in gastric fluid. On functional respiratory tests there was distal obstructive deficit with a normal DLCO. Bronchoscopy showed macroscopically normal bronchi. Bronchial biopsies suggested non specific inflammation lesions. Renal Ultrasound revealed poorly differentiated kidneys with a small left kidney, which didn't allow the renal biopsy. Skin biopsy was performed on healthy skin and showed no lupus band. Renal biopsy was contrindicated by the findings of the renal ultrasound. Biopsy of the salivary glands showed chronic sialadenitis the special Congo Redstain revealed amyloid deposits around the vessels and interlobular ducts (Figure 3) which are birefringent amyloid deposits under polarized light (Figure 4). Accordingly, the retained diagnosis was the association of SLE, sarcoidosis (Lofgren syndrome) and amyloidosis. Our patient has already been treated. There was a complete regression of the erythema nodosum lesions under analgesics and potassium iodide after 4 weeks. There were no indications for corticosteroid therapy because kidneys were already affected, CKD stage (Chronic kidney disease) with small dedifferentiated kidneys and there were no signs of activity of sarcoidosis. The patient was prescribed Colchicine^®^ 1mg per day and Nivaquine^®^ 200mg per day. The patient monitoring included a chest CT, a transthoracic echocardiography (which showed regression of the pericardial effusion), creatininemia and Proteinuria. A rectal biopsy was scheduled.

## Discussion

The association SLE and Sarcoidosis has been described in the literature as a non-fortuitous association [1]. The blocking of the reticuloendothelial purification system by immune complexes excess in SLE can enhance the formation sarcoid granulomas. Hepatitis C treatment with IFN-αcan induce sarcoidosis and SLE [2–4]. There is common cytokininic channel stimulation between the two conditions. Amyloidosis is defined by the extracellular deposition of protein agglomerates all having common tinctorial affinity, a fibrillary appearance in electron microscopy, and a so-called spatial conformation β-pleated sheet [5, 6]. There are several types of amyloidosis according to the nature of the deposits of amyloid proteins [7]. AA amyloidosis is a complication of chronic inflammation (Rhumatoid arthritis, Ankylosing spondylitis, Crohn's disease, Juvenile idiopathic arthritis) or chronic infections such as tuberculosis. Amyloidosis almost never occurs as a complication of SLE [8, 9]. There are a number of reasons which make LES and amyloidosis are mutually exclusive: there is low SAA protein levels during active lupus, whereas the developed amyloidosis secondary to SLE shows primarily elevated SAA protein levels (reactive amyloidosis) [10]. There have been nearly 30 reported cases that associate SLE and amyloidosis since 1956. It is most often an AA amyloidosis type. An AL amyloidosis has been described in some lupus with monoclonal gammopathy. The elapsed time from diagnosis of SLE and amyloidosis varies from 1year to 35 years. The diagnosis was concomitant in our patient. In half of the patients, amyloidosis appears in less than five years’ time. The occurrence of amyloidosis mechanisms is not very much known. High levels of SAA proteins were found in cases where they were assayed, which showed strong correlation between elevated SAA and amyloïdogenesis. The diagnosis of amyloidosis was confirmed according to histopathological findings after pulmonary, renal, gastric, cardiac and cutaneous biopsies. The association Sarcoidosis and Amyloidosis has been described in the literature. Five cases where reported in the literature with renal amyloidosis. The diagnosis of sarcoidosis in our case is to be discussed. The clinical features are highly suggestive of Lofgren's Syndrome, staged II on chest X-Ray. There no need to histological evidence as it is not mandatory

## Conclusion

Although concomitant SLE and amyloidosis is an exceptional combination it remains possible. Indeed amyloidosis can complicate any chronic inflammatory condition including SLE. It can be localized or generalized. Treatment is directly based on the management of the exacerbations of SLE
